# A new record of the genus *Mollitrichosiphum* Suenaga (Hemiptera, Aphididae, Greenideinae) from South Korea

**DOI:** 10.3897/BDJ.13.e157863

**Published:** 2025-10-01

**Authors:** Yejin Kang, Wonhoon Lee

**Affiliations:** 1 Department of Plant Medicine, Gyeongsang National University, Jinju, Republic of Korea Department of Plant Medicine, Gyeongsang National University Jinju Republic of Korea; 2 Institute of Agriculture & Life Science, Gyeongsang National University, Jinju, Republic of Korea Institute of Agriculture & Life Science, Gyeongsang National University Jinju Republic of Korea

**Keywords:** Greenideini, *
Mollitrichosiphum
*, new record, *COI*, *CytB*, South Korea

## Abstract

**Background:**

The genus *Mollitrichosiphum* Suenaga belongs to the subfamily Greenideinae, with 18 species hav been recorded worldwide.

**New information:**

The genus *Mollitrichosiphum* is a completely new record in South Korea. In 2024, Mollitrichosiphum (Metatrichosiphon) luchuanum (Takahashi, 1930) was collected on *Meliosma myriantha* located in Hawon-dong, Seogwipo-si, Jeju-do, South Korea (33.19590, 126.28340). Illustrations of apterous viviparous females and alate viviparous females, description, measurement, host plants and distributions are provided. In addition, DNA barcoding, based on a mitochondrial *cytochrome c oxidase subunit I* (*COI*) sequence and a mitochondiral *cytochrome b* (*CytB*) sequence are provided.

## Introduction

The subfamily Greenideinae (Hemiptera, Aphididae) consists of 16 genera including 180 species worldwide (Favret and Aphid Taxon Community, eds. 2025). The Greenideinae comprises three tribes: Cervaphidini van der Goot, 1917, Greenideini Baker, 1920 and Schoutedeniini Nieto Nafría, Mier Durante & Pérez Hidalgo, 2011 (Favret and Aphid Taxon Community, eds. 2025). Presently, three genera of two tribes except the Schoutedeniini have been reported in South Korea (Paik 1965, Paik 1972): Greenidea (Trichosiphum) kuwanai (Pergande, 1906), *Eutrichosiphum pasaniae* (Okajima, 1908), *Cervaphis quercus* Takahashi, 1918, and Greenidea (Trichosiphum) nipponica Suenaga, 1934.

The genus *Mollitrichosiphum* Suenaga 1934 is classified into two subgenera, *Mollitrichosiphum* and *Metatrichosiphon* (Remaudière and Remaudiėre 1997) and is mainly distributed in Southeast Asia (Zhang and Qiao 2010). *Mollitrichosiphum* species mainly feed on young leaves and branches of Fagaceae and *Meliosma* (Sabiaceae). In addition, several species colonizes other plants, including Betulaceae, Sabiaceae and Rosaceae (Zhang et al. 2012, Qin et al. 2021, Favret and Aphid Taxon Community, eds. 2025).

In this study, we report a new species, Mollitrichosiphum (Metatrichosiphon) luchuanum (Takahashi, 1930) for the first time in South Korea and provide morphological description with illustrations of apterous viviparous females and alate viviparous females that are found on *Meliosma myriantha*. In addition, the *COI* and *CytB* regions were used to analye phylogeny among closely related congeneric species.

## Materials and methods

**Sampling and morphological identification**

Colonies of Mollitrichosiphum (Metatrichosiphon) luchuanum (Takahashi, 1930) were collected from the undersides of the young leaves of *Meliosma myriantha* in Seogwipo Natural Recreational Forest, Seogwipo-si, Jeju-do, South Korea (33.19590, 126.28340) from 2024 to 2025. The samples were preserved in 95% alcohol and slide glass specimens were mounted on Canada balsam, following the method of Blackman and Eastop (2000) methods. Species identification was based on the morphological characteristics of apterous viviparous females, following the keys provided by Takahashi (1930), Zhang and Qiao (2010), Zhang et al. (2012), Jiang et al. (2015) and Favret and Aphid Taxon Community, eds (2025).

Examination of the specimens was taken by LEICA (DM3000 LED) and LEICA (CTR6 LED). All specimens were deposited in the Institute of Agriculture & Life Science, Gyeongsang National University, South Korea. The following abbreviations are used in morphological features: BL, body length from the head to the end of cauda; Ant., antennae; Ant.I–VI and Ant.VIb, antennal segments I–VI, and basal part of antennal segment VI, respectively; PT, processus terminalis; BDAnt.III, basal diameter of antennal segment III; Setae on Ant.Ⅲ, the longest seta on antennal segment III; 2HT, second segment of hind tarsus; HFM, hind femur; HTB, hind tibia; SIPH, siphunculus; SIPH BW, basal width of siphunculi; SIPH MW, maximum width of siphunculi; SIPH DW, distal width of siphunculi; Cauda BW, basal width of cauda; and URS, ultimate rostral segment.

**DNA extraction, amplification, sequence alignment and data analyses**

Total genomic DNA was extracted from one individual of apterous vivipara females using the AccuPrep® Genomic DNA Extraction Kit (Bioneer, Daejeon, South Korea), following the manufacturer’s protocol. The DNA fragments were amplified using AccuPower® PCR PreMix (Bioneer, Daejeon, South Korea). The partial sequences of the mitochondrial *COI* and *CytB* genes were amplified using the primer pairs: LCO1490 (5’-GGTCAACAAATCATAAAGATATTGG-3’)/HCO2198 (5’-TAAACTTCAGGGTGACCAAAAAATCA-3’) (Folmer et al. 1994) for the *COI* gene, and CP1(5′-GATGATGAAATTTTGGAT-3′) (Harry et al. 1998)/CB2(5′-ATTACACCTCCTAATTTATTAGGAAT-3′) (Jermiin and Crozier 1994) for the *CytB* gene. We used the following thermal cycle parameters for 20 μl amplification reaction: an initial denaturation for 5 min at 94°C, followed by 34 cycles of 1 min at 94°C, 1 min at 48°C and 1 min at 72°C and extension at 72°C for 5 min. The PCR products were tested by electrophoresis on 1.5% agarose gel. A single band was observed, purified using a QIAquick PCR purification kit (QIAGEN, Inc.), and directly sequenced using an automated sequencer (ABI PrismH 3730 XL DNA Analyzer) at MACROGEN LIC. The sequences were contiged using SeqMan (version 7.0.1, DNASTAR Inc.) and alignments were performed using MEGA version 12 (Kumar et al. 2024).

We compared our M. (M.) luchuanum sequence with other reference sequences of Mollitrichosiphum (Metatrichosiphon) nigrum, Mollitrichosiphum (Metatrichosiphon) rhusae in GenBank except unpublished sequences. Pairwise genetic distances were calculated using MEGA version 12 (Kumar et al. 2024) based on the Kimura 2 parameter (K2P) model (Kimura 1980). Neighbor-joining trees were also constructed with 1,000 bootstrapping replications replicates to assess node support (Saitou and Nei 1987). We selected *Greenidea kuwanai* as the outgroup, in comparison with the ingroup species, *M. rhusae*, *M. luchuanum*, and *M. nigrum* in this study.

## Taxon treatments

### Mollitrichosiphum (Metatrichosiphon) luchuanum

Takahashi, 1930

772356DE-3314-5D81-A4EB-66AC854E1A56

Greenidea luchuana - Takahashi 1930: 322
Mollitrichosiphum (Metatrichosiphon) luchuana - Ghosh 1974: 169
Mollitrichosiphum (Metatrichosiphum) luchuanum - Eastop and Hille Ris Lambers 1976: 209, 286
Mollitrichosiphum (Metatrichosiphon) luchuanum - Remaudière and Remaudiėre 1997: 177

#### Materials

- **Type status:** Other material. **Occurrence:** catalogNumber: coll#.20240619-001; recordedBy: Yejin Kang; individualCount: 12; sex: female; lifeStage: apterous female; occurrenceStatus: present; associatedOccurrences: host: Meliosma myriantha; occurrenceID: 75FCA5B7-39BF-5CB2-A182-87A018299BA2; **Taxon:** taxonID: Uncertain; scientificName: Mollitrichosiphum luchuanum; higherClassification: East Asia; kingdom: Animal; phylum: Arthropoda; class: Insecta; order: Hemiptera; family: Aphididae; genus: Mollitrichosiphum; subgenus: Metatrichosiphon; taxonomicStatus: accepted; **Location:** country: South Korea; countryCode: KR; stateProvince: Jeju-do; municipality: Seogwipo-si; locality: Hawon-dong; verbatimLatitude: 33.333056; verbatimLongitude: 126.476111; geodeticDatum: WGS84; georeferenceProtocol: label; **Identification:** identifiedBy: Yejin Kang; dateIdentified: 2024; **Event:** samplingProtocol: hand collected; year: 2024; month: 6; day: 19; **Record Level:** type: specimen; language: en; rightsHolder: Gyeongsang National University; collectionID: GNU; collectionCode: Insects; basisOfRecord: PreservedSpecimen
- **Type status:** Other material. **Occurrence:** catalogNumber: coll#.20250619-001; recordedBy: Yejin Kang; individualCount: 6; sex: female; lifeStage: alate female; occurrenceStatus: present; associatedOccurrences: host: Meliosma myriantha; occurrenceID: 383DCE44-92DC-5916-AEEF-F3A4E008614A; **Taxon:** taxonID: Uncertain; scientificName: Mollitrichosiphum luchuanum; higherClassification: East Asia; kingdom: Animal; phylum: Arthropoda; class: Insecta; order: Hemiptera; family: Aphididae; genus: Mollitrichosiphum; subgenus: Metatrichosiphon; taxonomicStatus: accepted; **Location:** country: South Korea; countryCode: KR; stateProvince: Jeju-do; municipality: Seogwipo-si; locality: Hawon-dong; verbatimLatitude: 33.333056; verbatimLongitude: 126.476111; geodeticDatum: WGS84; georeferenceProtocol: label; **Identification:** identifiedBy: Yejin Kang; dateIdentified: 2025; **Event:** samplingProtocol: hand collected; year: 2025; month: 6; day: 19; **Record Level:** type: specimen; language: en; rightsHolder: Gyeongsang National University; collectionID: GNU; collectionCode: Insects; basisOfRecord: PreservedSpecimen

#### Description

**Figure 1.**
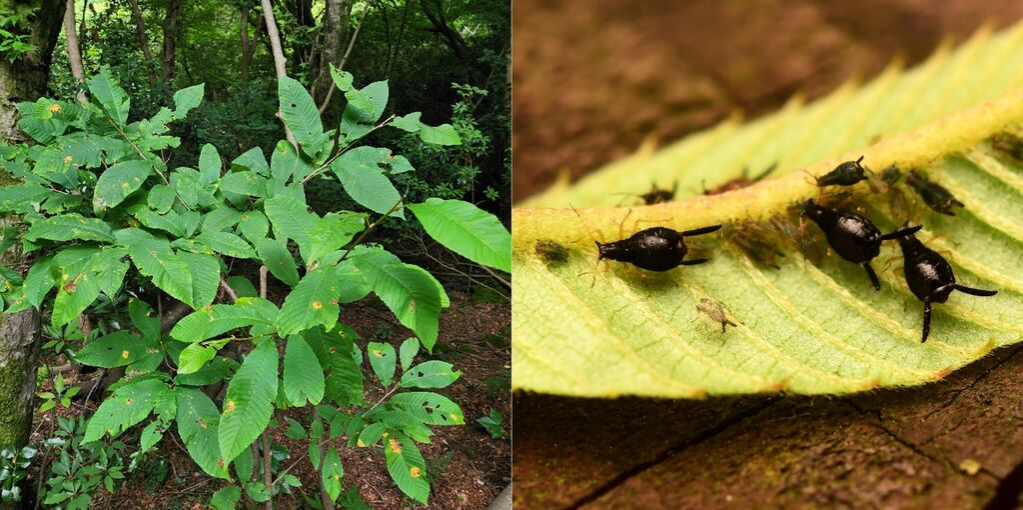
Apterous viviparous female of Mollitrichosiphum (Metatrichosiphon) luchuanum on *Meliosma myriantha* (coll#.20240619-001).

**Figure 2.**
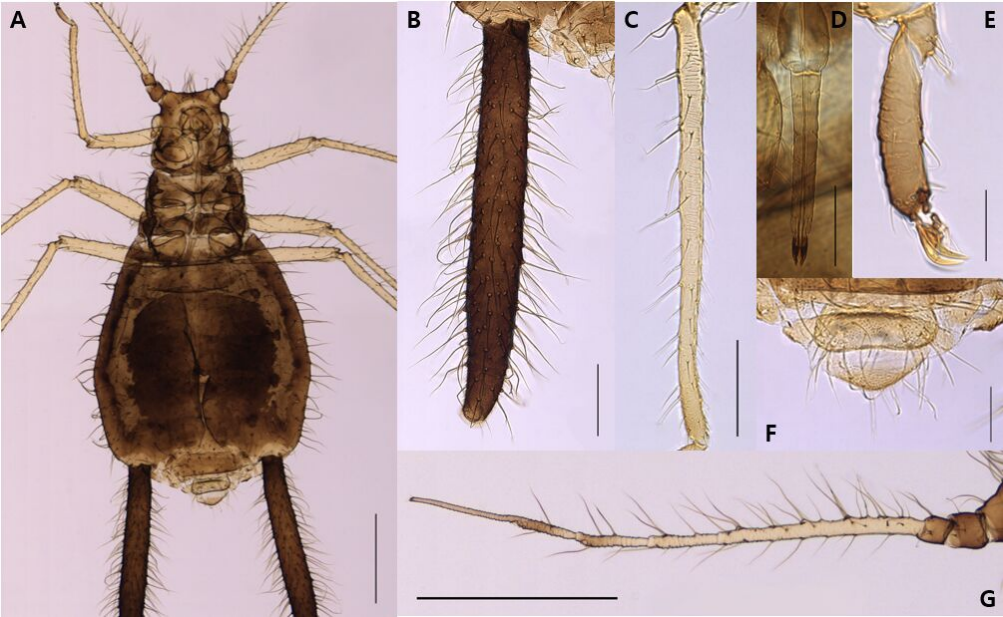
Apterous viviparous female of Mollitrichosiphum (Metatrichosiphon) luchuanum (coll#.20240619-001). **A**, Whole body; **B**, SIPH; **C**, HTB; **D**, URS; **E**, 2HT; **F**, Cauda; **G**, Whole Antenna. [Scale bar: 500㎛ (A, G); 200㎛ (B, C); 100㎛ (D, F); 50㎛ (E)]

**Apterous viviparous female** (Table 1). Body glossy black, leg yellowish brown, except each of the distal antennae segment and tarsi dark brown in life (Fig. 1). On mounted specimens, the body is dark brown oblong oval, 1.91-2.33mm long (Fig. 2A). Head is fused with prothorax; front 3 pairs of acuate setae, 3 or 4 pairs of dorsal setae between antennae, and 3 pairs of dorsal setae between eyes. Antennae 6-segmented, Ant.Ⅰ-Ⅱ, the end of Ⅴ-Ⅵb segment, and PT is brown; 1.19-1.54mm long (Fig. 2G). The long and short antennal setae are present together; the longest seta on antennal segment III is 0.18-0.21mm long. Ant.Ⅲ moderately transverse imbricated with 20-38 setae; Ant.Ⅳ-Ⅴ imbricated with 4-7, 4-6 setae; Ant.Ⅵ intensely imbricated with 3 or 4 setae on Ant.Ⅵb; PT 0.21-0.33mm long, with 4 short apical setae, 1.72-2.17 times as long as Ant.Ⅵb. End of rostrum passing the hind coxae; URS 0.23-0.27mm long, with 11-14 accessory setae (Fig. 2D). Hind femur is smooth, bearing slight spinules on the underside; hind tibia is smooth with 41-49 transverse ridges (Fig. 2C); 2HT brown, imbricated with 7-10 setae (Fig. 2E). Abdomen is spinulose ventrally; dorsal setae long and sharp, mainly pointed; ventral setae thin and fine-shaped. Siphunculus distinctly dark brown and long-shaped, bearing many long setae in general and spinulose on the both apical parts (Fig. 2B); 0.47-0.57 times as long as BL, 6.14-11.90 times as long as basal width of SIPH. Cauda nearly triangular with rounded apex, spinules dense with 7-9 setae (Fig. 2F); 0.40-0.45times as long as basal width of Cauda.

**Figure 3.**
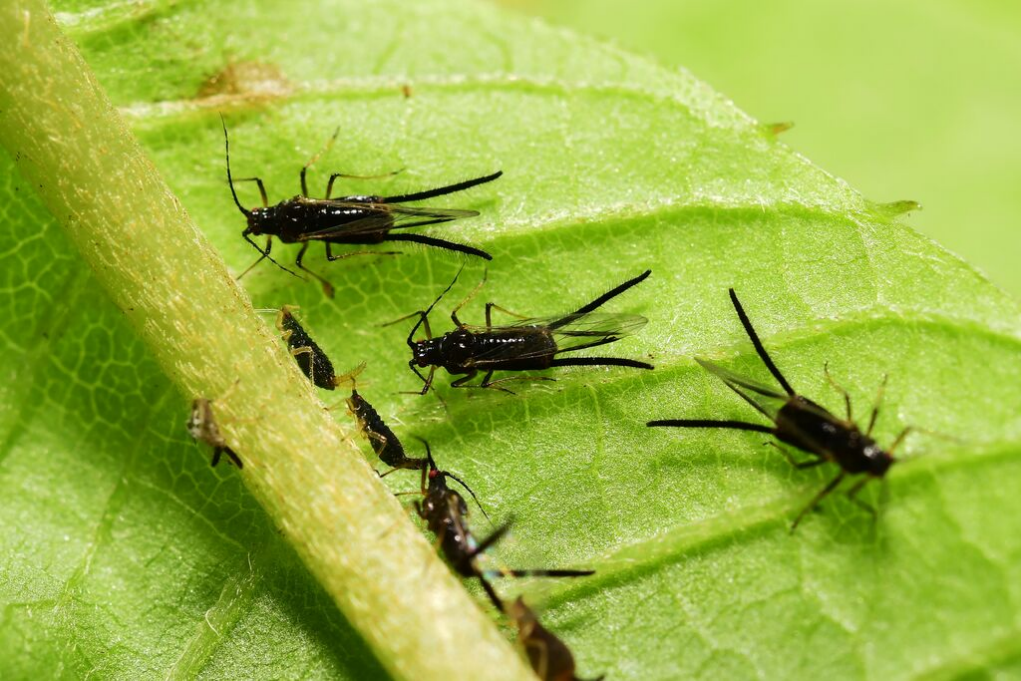
Alate viviparous female of Mollitrichosiphum (Metatrichosiphon) luchuanum (coll#.20250619-001).

**Figure 4.**
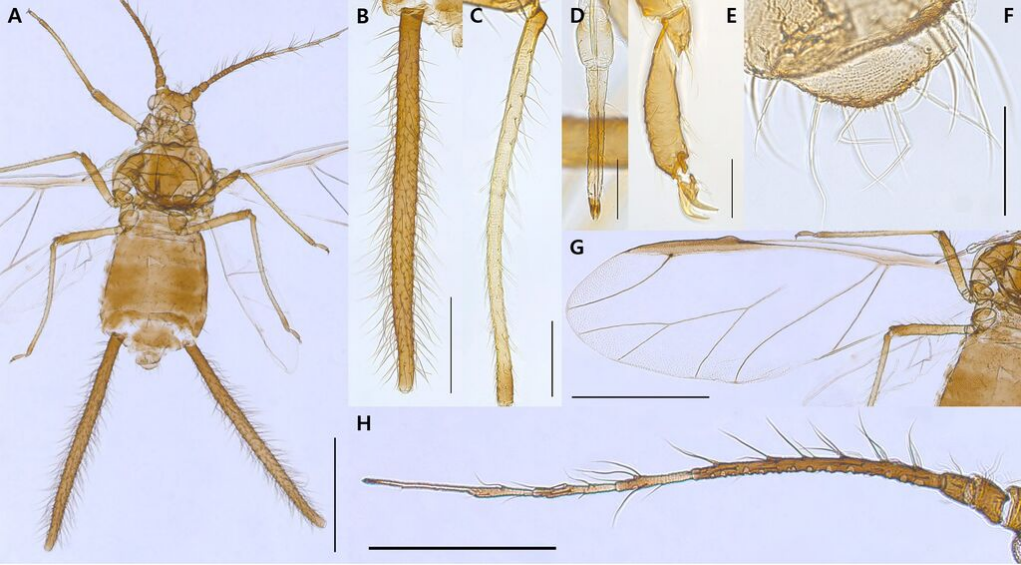
Alate viviparous female of Mollitrichosiphum (Metatrichosiphon) luchuanum (coll#.20250619-001). **A**, Whole body; **B**, SIPH; **C**, HTB; **D** URS; **E**, 2HT; **F**, Cauda; **G**, Fore wing; **H**, Whole Antenna. [Scale bar: 1mm (A, G); 500㎛ (B, H); 200㎛ (C); 100㎛ (D, F); 50㎛ (E)]

**Alate viviparous female** (Table 1). Body glossy black, forewing veins and siphunculi black with dense long setae and tibiae yellowish brown in life (Fig. 3). On mounted specimens, the body is brown oblong oval, 1.94-2.47mm long (Fig. 4A). Head is separated from prothorax. Antennae 6-segmented; Ant.Ⅰ-Ⅲ, the half of Ⅳ-Ⅵ segment, and the end of PT is brown; 1.51-1.77mm long (Fig. 4H). Antennal setae long and pointed, the longest seta on antennal segment III is 0.16-0.19mm long. Ant.Ⅲ with 17-22 round secondary rhinaria; Ant.Ⅳ-Ⅴ clearly transverse imbricated with 5-7, 5-6 setae; Ant.Ⅵ intensely imbricated with 3-4 setae on Ant.Ⅵb; PT 0.27-0.35mm long, with 4 short apical setae, 1.71-2.42 times as long as Ant.Ⅵb. URS 0.22-0.28mm long, with 15-19 accessory setae (Fig. 4D). Femur is brown, darker toward the tibiae; Hind femur with scattered spinules on the underside; hind tibia with 41-48 transverse ridges (Fig. 4C); 2HT is brown, imbricated (Fig. 4E). Veins of wings and pterostigma brown, fore wings bearing media twice branched; hind wings with 2 oblique veins (Fig. 4G). Abdomen is spinulose and fine-shaped setae, dorsal setae long and pointed. Siphunculus brown, significantly long and hairy with spinulose on basal and distal parts (Fig. 4B); 0.77-0.88 times as long as BL, 25.24-26.46 times as long as distal width of SIPH. Cauda semi-triangular, bearing spinules and 7-8 setae (Fig. 4F); 0.31-0.46 times as long as basal width of Cauda.

#### Distribution

South Korea (new record), Japan, China (Zhang and Qiao 2010, Favret and Aphid Taxon Community, eds. 2025).

#### Notes

It is known that this species inhabits *Meliosma* (Sabiaceae) in China (Zhang et al. 2012). In this study, we collected this species on the undersides of young leaves of *Meliosma myriantha* in South Korea (Fig. 1). Until now, their life cycle has not been revealed.

#### Host plants

*Meliosma myriantha* Siebold & Zucc. (in this study), *Meliosma rigida* Siebold & Zucc., *Prunus persica* (L.) Batsch and *Quercus* spp. (Raychaudhuri 1956, Zhang et al. 2012, Favret and Aphid Taxon Community, eds. 2025).

## Identification Keys

### Identification key to species of the subfamily Greenideinae in South Korea

**Table:** 

1	Body pale yellow, bearing many elongated branched projections. SIPH elongated, with short setae	*Cervaphis quercus*
–	Body brown, bearing long and short setae without elongated branched projections. SIPH plump, with long setae	2
2	5-segmented antennae. SIPH not reticulated	*Eutrichosiphum pasaniae*
–	6-segmented antennae. SIPH bearing reticulated only at the base	3
3	Abdomen smooth ventrally	Greenidea (Trichosiphum) nipponica
–	Abdomen intensely spinulous ventrally	4
4	Hind tibia without transverse ridges, dark brown on basal (1/3) and pale brown on distal (2/3)	Greenidea (Trichosiphum) kuwanai
–	Hind tibia with many transverse ridges, pale brown usually	Mollitrichosiphum (Metatrichosiphon) luchuanum

## Analysis

**Figure 5.**
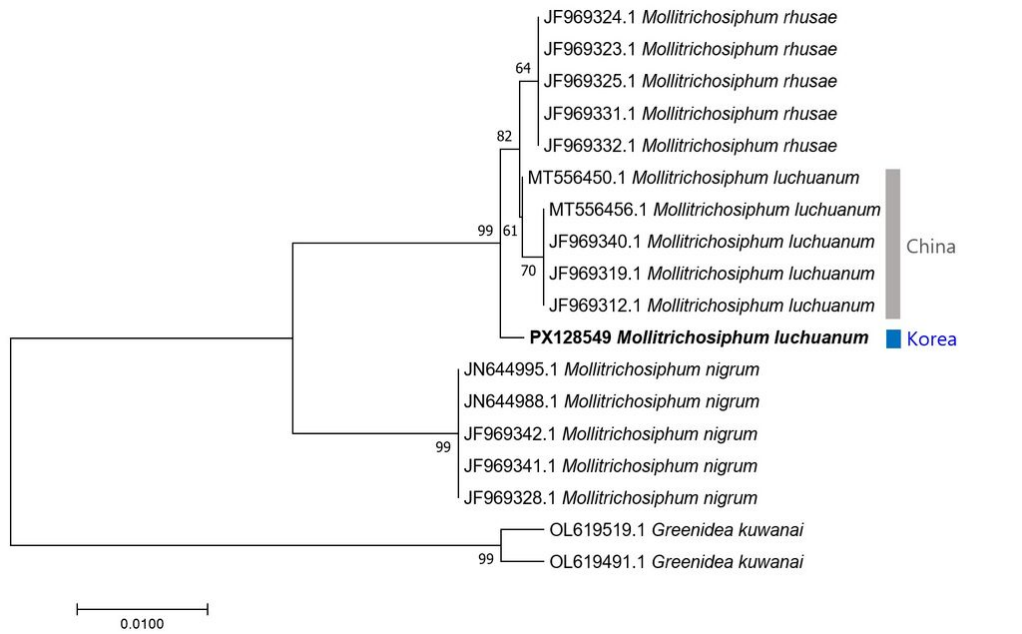
A neighbor–joining tree of *Mollitrichosiphum* spp. using *COI* sequence data. G. (T.) kuwanai was used as an outgroup. Bootstrap support values are displayed on each node.

**Figure 6.**
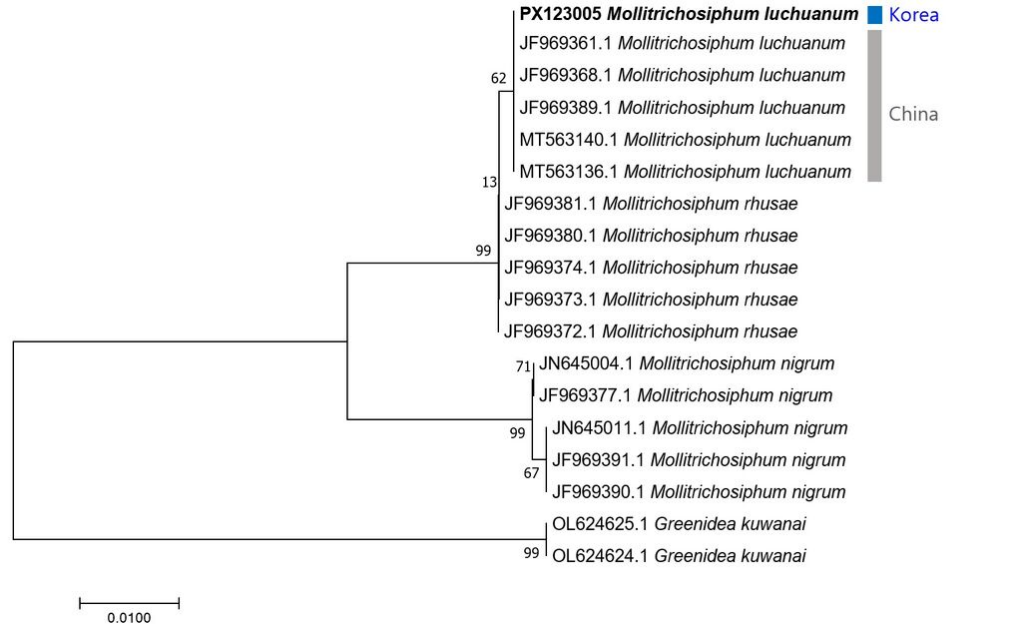
A neighbor–joining tree of *Mollitrichosiphum* spp. using *CytB* sequence data. G. (T.) kuwanai was used as an outgroup. Bootstrap support values are displayed on each node.

Two neighbor–joining trees, based on K2P genetic distances, were constructed with eighteen sequences from four species, including an outgroup (Figs 5, 6). In the *COI* dataset, M. (M.) luchuanum (PX128549) was separated from previously reported M. (M.) luchuanum sequences (Zhang et al. 2012, Jiang et al. 2015, Qin et al. 2021), whereas in the *CytB* dataset, the Korean specimen (PX123005) clustered within them (Zhang et al. 2012, Jiang et al. 2015). K2P distances between the Korean and previously reported M. (M.) luchuanum sequences were 0.3–0.5% (*COI* gene) and 0% (*CytB* gene), respectively.

## Supplementary Material

XML Treatment for Mollitrichosiphum (Metatrichosiphon) luchuanum

## Figures and Tables

**Table 1.** The biometric data of Mollitrichosiphum (Metatrichosiphon) luchuanum.

Body parts		Apterous viviparous female (n=12)		Alate viviparous female (n=6)	
		Mean	Range	Mean	Range
Length (mm)	Body	2.21	1.91–2.33	2.24	1.94–2.47
	Body width	1.10	0.92–1.21	0.92	0.83–1.00
	Head width	0.44	0.40–0.46	0.45	0.44–0.47
	Whole Antennae	1.44	1.19–1.54	1.65	1.51–1.77
	Ant.Ⅰ	0.08	0.07–0.09	0.09	0.09–0.10
	Ant.Ⅱ	0.07	0.06–0.08	0.08	0.07–0.08
	Ant.Ⅲ	0.51	0.40–0.56	0.62	0.53–0.68
	Ant.Ⅳ	0.17	0.15–0.18	0.20	0.18–0.21
	Ant.Ⅴ	0.19	0.16–0.21	0.21	0.18–0.23
	Ant.Ⅵb	0.15	0.12–0.16	0.16	0.14–0.18
	PT	0.29	0.21–0.33	0.31	0.27–0.35
	HFM	0.54	0.44–0.59	0.56	0.47–0.62
	HTB	0.89	0.75–0.94	0.98	0.89–1.05
	2HT	0.13	0.12–0.14	0.12	0.10–0.13
	URS	0.25	0.23–0.27	0.25	0.22–0.28
	SIPH	1.18	0.97–1.32	1.86	1.67–2.03
	SIPH BW	0.12	0.10–0.20	0.11	0.10–0.12
	SIPH MW	0.15	0.13–0.17	0.13	0.11–0.14
	SIPH DW	0.08	0.08–0.10	0.07	0.06–0.08
	Cauda	0.07	0.07–0.08	0.07	0.06–0.08
	Cauda BW	0.18	0.16–0.19	0.18	0.17–0.23
	BDAnt.Ⅲ	0.04	0.04–0.05	0.04	0.04–0.05
	Setae on Ant.Ⅲ	0.20	0.18–0.21	0.17	0.16–0.19
No. of setae on	Ant.Ⅰ	6	5–7	5	4–7
	Ant.Ⅱ	4	3–5	4	3–5
	Ant.Ⅲ	33	20–38	28	23–32
	URS	13	11–14	17	15–19
	Cauda	8	7–9	7	7–8
Ratio (times)	Whole Ant/BL	0.65	0.58–0.70	0.74	0.70–0.77
	PT/Ant.Ⅵb	1.95	1.72–2.17	1.90	1.71–2.42
	PT/Ant.Ⅲ	0.56	0.52–0.64	0.50	0.47–0.56
	URS/2HT	1.95	1.78–2.08	2.18	2.02–2.31
	URS/Ant.Ⅵb	1.72	1.61–1.89	1.56	1.36–1.72
	HFM/Ant.Ⅲ	1.06	0.97–1.24	0.91	0.85–1.02
	SIPH/BL	0.53	0.47–0.57	0.83	0.77–0.88
	SIPH/Ant.Ⅲ	2.30	2.18–2.44	3.02	2.87–3.20
	SIPH/SIPH BW	10.01	6.14–11.90	16.88	15.31–18.10
	SIPH/SIPH MW	7.69	7.24–8.97	14.84	14.31–15.27
	SIPH/SIPH DW	14.43	12.05–17.12	25.87	25.24–26.46
	Cauda/ Cauda BW	0.42	0.40–0.45	0.40	0.31–0.46
	Setae on Ant.Ⅲ/BDAnt.Ⅲ	4.75	4.12–5.47	3.97	3.73–4.38
